# Diagnostic accuracy of a simplified minimally invasive tissue sampling protocol for stillbirths in low-resource settings

**DOI:** 10.1136/bmjgh-2024-018380

**Published:** 2025-10-28

**Authors:** Núria Peñuelas, Adela Saco, Juan Carlos Hurtado, Lorena Marimon, Rosauro Varo, Inacio Mandomando, Jessica Navero-Castillejos, Carla Carrilho, Fabiola Fernandes, Assucena Guisseve, Mamudo R Ismail, Alba Morató, Cesaltina Lorenzoni, Aida Tivane, Laia Diez-Ahijado, Jesus Lopez-Diaz, Katarzyna Darecka, Tacilta Nhampossa, Quique Bassat, Mikel Martinez, Clara Menéndez, Raquel Gonzalez, Jaume Ordi, Natalia Rakislova

**Affiliations:** 1 ISGlobal, Barcelona, Spain; 2 Facultat de Medicina, Universitat de Barcelona, Barcelona, Spain; 3 Department of Pathology, Hospital Clínic de Barcelona, Barcelona, Spain; 4 Department of Microbiology, Hospital Clínic de Barcelona, Barcelona, Barcelona, Spain; 5 Centro de Investigação em Saúde de Manhiça, Manhiça, Mozambique; 6 Department of Pathology, Maputo Central Hospital, Maputo, Mozambique; 7 Department of Pathology, Universidade Eduardo Mondlane, Maputo, Mozambique; 8 ICREA, Barcelona, Pg. Lluís Companys 23, 08010, Spain; 9 Consorcio de Investigación Biomédica en Red de Epidemiología y Salud Pública, Madrid, Spain

**Keywords:** Other diagnostic or tool, Global Health, Health economics, Obstetrics

## Abstract

**Background:**

Stillbirth rates remain unacceptably high in low- and middle-income countries (LMICs). Understanding the causes of death (CoD) is mandatory to develop effective strategies to reduce this high mortality. Minimally invasive tissue sampling (MITS) is a promising alternative to conventional autopsy (CA) but its validation in stillbirths remains limited. Existing evidence indicates that most samples of conventional MITS (c-MITS) lack diagnostic relevance in stillbirths. This study aimed to validate c-MITS against CA in stillbirths and design and assess a cost-efficient, simplified MITS (s-MITS) protocol.

**Methods:**

The study comprised two subsets of stillbirths occurring at Maputo Central Hospital, Mozambique. The CaDMIA-Plus cohort (*Cause of Death investigation using Minimally Invasive Autopsy*, n=90; 2017–2018), in which both c-MITS and CA were performed, was used to validate c-MITS against the gold standard and to determine the diagnostic value of each sample and design a s-MITS. The MIBio cohort (*Mortality Identification Biomarkers*, n=98; 2021–2022), which included only s-MITS, was used to evaluate the performance and cost of the s-MITS in comparison to c-MITS in an independent cohort.

**Results:**

Almost perfect overall agreement (Kappa=0.82) was observed between the c-MITS and CA-attributed CoD. Lung and placenta samples were identified as the most informative in c-MITS. When using only lung and placenta results to model an s-MITS, substantial agreement (Kappa=0.79) was found between the s-MITS-derived CoD and those attributed by CA. Similar CoD distributions were observed when applying the s-MITS to the MIBio cohort, while costs were reduced by 55.7%. The leading CoDs were primarily related to maternal conditions and pregnancy complications (70.0–72.4%) and infectious diseases (25.6–27.6%).

**Conclusions:**

c-MITS is a simpler and cost-effective alternative to CA for determining CoD in stillbirths. s-MITS has a diagnostic accuracy similar to that of c-MITS, while significantly reducing costs, making it adequate for implementation in routine clinical practice in LMICs.

**WHAT IS ALREADY KNOWN ON THIS TOPIC**

- Accurate understanding of the causes of death (CoD) is mandatory to develop effective strategies to reduce mortality.
- Minimally invasive tissue sampling (MITS), also known as minimally invasive autopsy, is a feasible and less invasive alternative to conventional autopsy (CA) for determining CoD in various age groups, including stillbirths, neonates and children.
- Previous studies, including the CaDMIA project (*Cause of Death investigation using Minimally Invasive Autopsy*), have shown that MITS provides accurate CoD diagnoses with significant concordance with CA in stillbirths. However, these studies were limited by small sample sizes (n=15 and n=18).
- MITS remains costly, particularly in stillbirths, in which non-essential organs and extensive laboratory testing tend to yield low diagnostic value.
- This resource-intensive nature limits the widespread adoption of MITS in low- and middle-income countries (LMICs), where accurate information on CoD is most needed.

**WHAT THIS STUDY ADDS**

- This study, the largest cohort to date validating conventional MITS (c-MITS) against CA in stillbirths, confirms the high diagnostic accuracy of MITS.
- The lungs and the placenta were the most diagnostically relevant organs in stillbirths. A simplified MITS protocol (s-MITS), focused only on these two organs, was designed.
- The s-MITS provided comparable diagnostic accuracy to c-MITS while reducing costs by 55.7%, showing that s-MITS is an accurate, cost-effective and scalable alternative to c-MITS.

**HOW THIS STUDY MIGHT AFFECT RESEARCH, PRACTICE, OR POLICY**

- This study shows that the s-MITS reduces time, resources and costs while maintaining high diagnostic accuracy and thus, it is particularly suitable for implementation in LMICs.
- The s-MITS can provide information for developing health policies and has the potential to improve surveillance of causes of stillbirth in LMICs.

##  Introduction

In 2023, an estimated 1.9 million babies were stillborn globally.^1^ Stillbirth rates remain deeply unequal across countries, with sub-Saharan Africa accounting for 50% of the global total and South Asia for 31%.^1^ While there has been some progress over the past two decades—with the global stillbirth rate falling from 22.6 per 1000 total births in 2000 to 14.3 in 2023, representing a 37% reduction—this improvement has lagged behind that reported for childhood and neonatal mortality.^1^,^3^ Indeed, in many sub-Saharan African countries, the number of stillbirths has shown little to no improvement or even increased, as population growth outpaced reductions in the stillbirth rate.^1^ The ‘Every Woman, Every Newborn Everywhere’ Plan set a goal for all countries to reduce stillbirth rates to ≤12 deaths per 1000 total births by 2030 and address equity gaps, though 52 countries—43 of them in sub-Saharan Africa—are currently off track to meet this target.^4^

An accurate understanding of the causes of death (CoD) is a mandatory prerequisite to develop effective strategies to reduce this burden.^2^ Conventional autopsy (CA) remains the gold standard for determining CoD, but performing these examinations in low- and middle-income countries (LMICs) is challenging due to cultural, logistical and resource barriers.^5^ Consequently, CoD data in these regions often rely on verbal autopsy and/or clinical records, which are highly inaccurate, particularly in determining stillbirth CoD.^6^,^8^

Minimally invasive tissue sampling (MITS), also known as minimally invasive autopsy, is an alternative to CA and is based on performing a series of percutaneous punctures to obtain fluids and tissue samples from key organs, which are analysed using pathological and microbiological techniques.^9^,^11^ MITS has gained recognition as a feasible postmortem examination method due to its shorter procedure time, reduced need for technical and human resources and less invasive nature, making it more acceptable than CA, especially in LMICs.^12^

Over the past decade, the pioneering CaDMIA project (*Cause of Death investigation using Minimally Invasive Autopsy*) showed that MITS in LMICs has excellent concordance with CA in determining CoD across different age groups, including adults,^13 14^ children,^15^ stillborn babies and neonates.^16^ Several studies have further validated its feasibility and accuracy in diverse LMICs with different sociocultural backgrounds and epidemiological characteristics, particularly highlighting its diagnostic accuracy in children and neonates.^17^,^19^ The Child Health and Mortality Prevention Surveillance (CHAMPS), a global health initiative aimed at systematically collecting data from under-five childhood deaths and monitoring CoD in LMICs,^20^ has been routinely employing MITS across seven sites in sub-Saharan Africa and South Asia.^21^ In spite of the increasing use of MITS in stillbirth mortality research surveillance,^22^,^26^ studies that rigorously validate its accuracy against CA in this age group are limited, with only two studies involving cohorts of 18 and 15 cases, respectively.^16 19^

In addition to accuracy, cost is critical when considering the implementation of any procedure, particularly in LMICs.^27^ A study performed in an academic hospital in the Netherlands reported a total cost of €1296 per MITS, including the materials used and personnel involved.^28^ Another study, evaluating the cost of conducting MITS in four LMICs, reported an approximate cost per case ranging from $609 to $1028.^29^ Therefore, optimising MITS is crucial, particularly in stillbirths, in which several samples and tests yield low diagnostic value.^30^

This study aimed to: (1) validate conventional MITS (c-MITS) against the gold standard CA, in a large cohort of stillbirths; (2) identify the most relevant organs and tests for determining the CoD, and design a simplified MITS (s-MITS) based on this information; and (3) evaluate the performance and cost of the s-MITS in comparison to c-MITS in an independent cohort.

## Methods

### Study design and setting

This study included two stillbirth cohorts from two projects: (1) CaDMIA-Plus (*Cause of Death investigation using Minimally Invasive Autopsy-Plus*), a project that included stillbirths occurring between February 2017 and August 2018, aimed at validating c-MITS against CA and designing a s-MITS protocol by evaluating the diagnostic value of the different samples and tests; and (2) MIBio (*Mortality Identification Biomarkers*), a project that included stillbirths occurring from March 2021 to April 2022, aimed at evaluating the feasibility, cost and performanceof the s-MITS protocol.

The two studies were conducted at the Departments of Obstetrics and Gynaecology and Pathology of the Maputo Central Hospital (MCH), a quaternary 1500-bed government-funded institution that serves as the referral centre for hospitals in southern Mozambique.

The inclusion criteria for both cohorts were: (1) perinatal deaths meeting the WHO definition of stillbirth: babies born with no signs of life after 22 gestational weeks or with a weight greater than 500 g^31^; and (2) written informed consent from the relatives.

### Revision of the clinical records

For all the cases included in the study, the maternal clinical records were carefully reviewed and abstracted into standardised data collection questionnaires. Demographic information, as well as antenatal and obstetric data, was extracted by an obstetrician and recorded using REDCap electronic data capture tools hosted at Barcelona Institute for Global Health (ISGlobal).^32 33^

### Postmortem procedures

**Figure 1:**
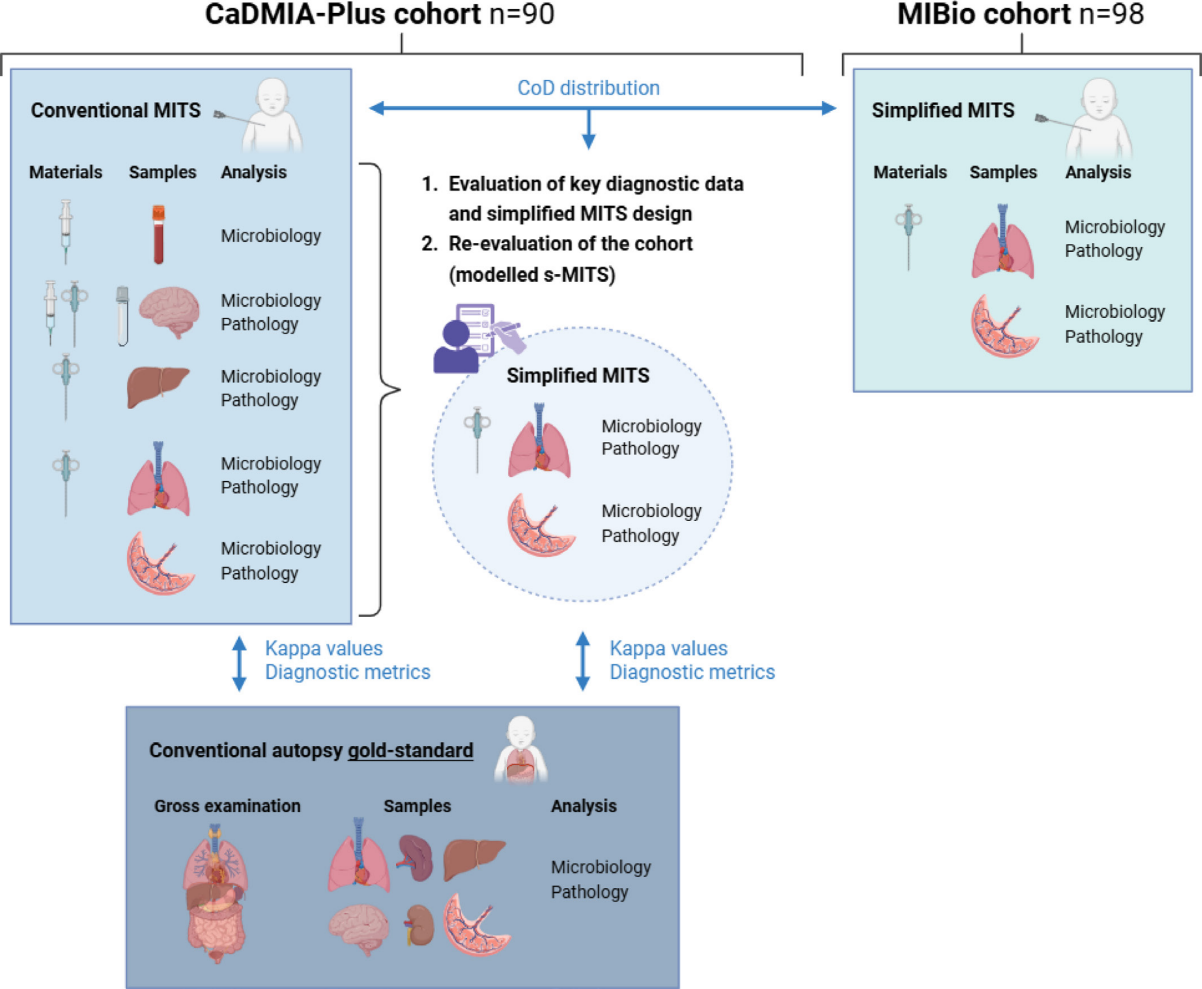
Diagram illustrating the study flow. In the CaDMIA-Plus cohort (n=90), conventional minimally invasive tissue sampling (c-MITS) and the gold standard conventional autopsy (CA) were conducted. Causes of death (CoD) were assigned based on these two postmortem procedures (c-MITS and CA). After evaluating the diagnostic information provided by each sample and test, a simplified MITS (s-MITS) was designed, focusing only on the most diagnostically relevant samples from c-MITS (ie, lungs and placenta). CoD was then assigned based on only these samples, modelling the s-MITS approach. Kappa values and diagnostic performance metrics for determining CoD were calculated for conventional MITS (c-MITS) and simplified (s-MITS) compared with the CA in the CaDMIA-Plus cohort. s-MITS was then performed in the MIBio cohort (n=98), and CoD distribution between CaDMIA-Plus (using c-MITS) and MIBio cohort (using s-MITS) was compared. CaDMIA-Plus, *Cause of Death investigation using Minimally Invasive Autopsy-Plus;* MIBio*, Mortality Identification Biomarkers.*

All postmortem procedures were conducted at the Pathology Department. A diagram detailing the main materials used and the samples collected for the various postmortem procedures is shown in figure 1.

#### Common procedures (CaDMIA-Plus and MIBio cohorts)

Each fetus was weighed and anthropometric measurements, including body length, head circumference, thoracic and abdominal circumferences, right foot and right leg lengths, were taken. A thorough external inspection was conducted to rule out congenital anomalies, trauma, skin lesions and maceration. Photographs of front and back sides and nails were taken. The body was carefully cleaned and disinfected.

The fresh placenta was photographed, measured and weighed. Fresh samples, from both the maternal and fetal sides of the placental disk, membranes and umbilical cord were collected for microbiology. A blood sample was also collected from the parenchyma on the maternal side of the placenta. The placental disk, membranes and cord were then immersed in formalin for 24–72 hours. The fixed placenta was examined and sampled in accordance with the recommendations of the Amsterdam Placental Workshop Consensus.^34^

#### Conventional MITS (CaDMIA-Plus cohort)

The c-MITS was conducted following the protocol of the CaDMIA study.^11 35^ In addition to the common procedures described above, 10 mL of blood and cerebrospinal fluid (CSF) were collected for microbiology with a 20G spinal puncture needle via subclavian vein and occipital punctures, respectively. The main fetal organs (liver, both sides of the thorax [lungs and heart]) and central nervous system [CNS]) were sampled using a new 16G biopsy needle for each organ to avoid cross-contamination, which is critical for microbiological testing. Following the collection of microbiological samples, samples for histology were taken from the same organs with the same biopsy needle. The liver and thorax (targeting both lungs and heart) were sampled with two 16G needles (Bard Monopty), whereas the CNS was sampled using a 16G needle (Biomol) via transfontanelle (anterior and posterior) and through a transnasal approach.

#### Conventional autopsy (CaDMIA-Plus cohort)

Immediately after c-MITS, a CA was performed by another pathology team on the same body. A dissection with gross evaluation of all organs was conducted.^36^ Samples from the same viscera collected in the c-MITS, as well as from any grossly identified lesions, were obtained.

### Pathological processing and evaluation

Histological samples from both cohorts were processed in the Pathology Laboratory of the MCH.^11^ Briefly, tissue specimens were fixed in 10% neutral buffered formalin for 4 hours and embedded in paraffin using routine protocols. Four-micron sections were stained with H&E. Histochemical and/or immunohistochemical stains were used as needed.

### Microbiological testing

#### Common microbiological testing (CaDMIA-Plus and MIBio cohorts)

A basic screening was performed on all lung and placental samples, including PCR for TORCH infections (toxoplasma, herpes simplex virus 1 and 2, varicella-zoster virus, *T. pallidum*, Parvovirus 19, cytomegalovirus, enterovirus, rubella and lymphocytic choriomeningitis virus), PCR for *S. agalactiae,* and generic 16S-rRNA PCR for other bacteria. HIV-1/2 serologies were performed in plasma extracted from placental blood.

#### Conventional microbiological testing (CaDMIA-Plus cohort)

TORCH screening was also applied to the CSF, plasma and CNS samples. In plasma, *Plasmodium falciparum* and *T. pallidum* were analysed by PCR, and HIV-1/2 and hepatitis B and C antibodies were screened, along with viral load (if applicable). Lung, CNS, liver, placenta (including membranes) and blood were also tested by PCR for specific bacteria associated with sepsis (*S. agalactiae*, *Neisseria meningitidis, S. pneumoniae*, *Haemophilus influenzae*, *Escherichia coli*, *Listeria monocytogenes* and *Mycobacterium tuberculosis*). CNS samples were additionally tested for lymphocytic choriomeningitis virus. Bacterial and fungal cultures were performed in blood, liver, lungs and CNS samples.

### CoD determination and identification of key diagnostic samples and tests (c-MITS and CA)

A panel composed of a paediatrician, an obstetrician, a pathologist and a microbiologist assessed all the available information for each case (clinical records, photographs, anthropometric measurements and laboratory results). The main maternal and main fetal CoD were assigned using the WHO International Classification of Diseases for Perinatal Mortality (ICD-10 PM).^37^ The specific CoDs were grouped into broader categories for analysis, including pregnancy and fetal development alterations, infectious and non-conclusive cases, in which neither a maternal nor a fetal CoD could be identified.

In the CaDMIA-Plus study, c-MITS results were discussed first. Then, a separate expert panel, blind to the c-MITS procedure and findings, reviewed each CA result and assigned the CoD for each case.

During these panel meetings, the type of data (ie, clinical history, pathological and microbiological) and the sampled organ crucial for determining the CoD for each case were identified. For each crucial organ, the most relevant laboratory result was assigned (pathology, microbiology or both).

### Evaluation of the diagnostic information provided by each sample and test and design of a simplified MITS

Using the information of the CaDMIA-Plus study, the results provided by each sample and diagnostic test were carefully evaluated. Based on this information, the s-MITS protocol, including only the most informative samples and tests, was designed.

### Simplified MITS procedure (MIBio cohort)

The s-MITS included the common procedures for external body inspection and placental evaluation. However, the sampling included only both sides of the thorax (lungs and heart), using a 16G biopsy needle (Bard Monopty) for microbiological and histological analyses. Only one 16G biopsy needle was used for tissue sample collection. CSF, fetal blood, CNS and liver samples were not collected.

### CoD determination (s-MITS)

For the MIBio cohort, a panel composed of a paediatrician, an obstetrician, a pathologist and a microbiologist assessed all the available information for each case from the samples obtained in the s-MITS and assigned the CoD. For the CaDMIA-Plus cohort, a different expert panel, blind to the c-MITS and CA procedure and findings, and having only access to the pathological and microbiological data of the lungs and the placenta, in addition to the clinical data, assigned the CoD.

### Cost of a conventional and a simplified MITS

The costs of c-MITS and s-MITS, including materials for sample collection and analytical expenses, were compared by considering all supplies included in the MITS kit and the analytical costs within this specific study setting.

### Data management and analysis

All the study data were managed using REDCap electronic data capture tools hosted at ISGlobal.^32 33^ Statistical analyses were performed using R (V.4.4.0). We used absolute numbers and percentages to summarise qualitative data, and medians and IQRs for quantitative variables. Wilcoxon rank sum tests, χ² tests or Fisher’s exact tests were conducted based on the data requirements, to evaluate differences in maternal and fetal descriptive variables and CoD distribution between the cohorts. Kappa analysis was performed to assess the agreement between c-MITS and s-MITS with the gold standard CA in determining the CoD, including CoD category, main fetal CoD and main maternal CoD. Kappa values were interpreted following guidelines by Landis and Koch.^38^ Sensitivity, specificity, positive predictive value and negative predictive value were calculated for c-MITS and s-MITS compared with the gold standard CA.

## Results

### General characteristics of the cases included

In the CaDMIA-Plus cohort, a total of 90 stillbirths were included (median gestational age: 34.0 weeks (IQR 30–38 weeks); 54% male, 46% female). In the MIBio cohort, a total of 98 stillbirths were included (median gestational age: 34.4 weeks (IQR 31.1–37.3 weeks); 64% male, 36% female). The maternal and fetal variables for the cases enrolled in each cohort are shown in table 1. Significant differences were detected in the mother’s origin (urban vs rural), type of delivery (vaginal vs caesarean section), HIV status, antibiotic use during pregnancy or delivery and postmortem interval (time from death to MITS).

**Table 1:** Demographic and clinical maternal and fetal characteristics of the cases included in the CaDMIA-Plus and MIBio cohorts.

	Conventional MITS (CaDMIA-Plus, n=90)	Simplified MITS (MIBio, n=98)	P value^*^
Maternal characteristics			
Mother’s origin			**<0.001**
Urban	84 (93.3)	63 (64.3)	
Rural	6 (6.7)	35 (35.7)	
Mother’s age (years)	28 (23–32)	29 (24–34)	0.263
Maternal death			0.173
Yes	2 (2.2)	7 (7.1)	
No	88 (97.8)	91 (92.9)	
Gravidity			0.727
<3	39 (43.3)	46 (46.9)	
≥3	51 (56.7)	52 (53.1)	
Type of delivery			**<0.001**
Vaginal	58 (64.4)	87 (88.8)	
Caesarean section	32 (35.6)	11 (11.2)	
HIV status			**<0.001**
Negative	49 (54.4)	82 (83.7)	
Positive	6 (6.7)	16 (16.3)	
ND^†^	35 (38.9)	0 (0.0)	
Stained amniotic fluid			0.677
Yes	5 (5.6)	8 (8.2)	
No	85 (94.4)	90 (91.8)	
Antibiotics during pregnancy or delivery			**<0.001**
No	80 (88.9)	53 (54.1)	
Yes	10 (11.1)	45 (45.9)	
Fetal characteristics			
Gestational age (weeks)	34.0 (31.0–38.0)	34.4 (31.1–37.3)	0.892
Unknown	30	0	
*Sex*			0.221
Male	49 (54.4)	63 (64.3)	
Female	41 (45.6)	35 (35.7)	
Fetal weight (grams)	1.985 (1.235–2.600)	1.698 (1.188–2.473)	0.403
*Maceration*			0.230
Fresh	37 (41.1)	31 (31.6)	
Macerated	53 (58.9)	67 (68.4)	
Postmortem interval (hours)	27 (18–50)	46 (22–70)	**0.002**

Categorical variables are summarised as total counts and percentages. Continuous variables are summarised using the median and IQR.

Bold P values indicate statistically significant differences (P <0.05).

* Wilcoxon rank-sum test was performed for continuous variables; Pearson’s χ² test was performed for categorical variables; Fisher’s exact test was performed for the variable *maternal death*.

† The amount of plasma in these cases was insufficient to conduct the testing. ND; Not determined.

CaDMIA-Plus, Cause of Death investigation using Minimally Invasive Autopsy-Plus; MIBio, Mortality Identification Biomarkers; MITS, minimally invasive tissue sampling.

### Validation of c-MITS against the gold standard CA (CaDMIA-Plus cohort)

The concordance between the CoD assigned by c-MITS and those determined by CA in the CaDMIA-Plus study is shown in online supplemental table S1. Complete agreement was observed in 83 out of 90 cases for the main CoD categories, including pregnancy and fetal development alterations, infectious and non-conclusive causes. The overall concordance for the CoD category was almost perfect, with a Kappa value of 0.82 (95% CI 0.70 to 0.94, p<0.001).

The diagnostic performance of c-MITS compared with the gold-standard CA is shown in online supplemental table S2. The sensitivity and specificity of c-MITS for diagnosing CA-determined CoD ranged across the different CoD categories from 0.82 to 0.97 and from 0.89 to 1.00, respectively.

### Essential data to determine the CoD in the conventional MITS (CaDMIA-Plus cohort): design of a s-MITS protocol

In the 86 cases with conclusive CoD, both clinical and pathological data were crucial in 31 cases (36.0%), pathology alone in 30 cases (34.9%), clinical data alone in 19 cases (22.1%), pathology and microbiology in 6 cases (7.0%), whereas microbiology alone or combined with clinical data was key in none of the cases (0.0%).

Among the 67 cases in which laboratory results (pathology and microbiology) were crucial, only two organs, the lungs and the placenta, were deemed useful for CoD attribution. Laboratory results of the CNS, liver, CSF and blood were not essential for determining the final CoD in any of the cases. Examination of both the placenta and lungs was essential in 53 cases (79.1%), with the pathology of both organs alone being sufficient in most cases (45 cases; 84.9%), and in the remaining cases, microbiology was also required, either from both organs (7 cases) or exclusively from the placenta (1 case). In the remaining 14 cases (20.9%), examination of only one organ, either the lungs or the placenta, was sufficient to assign CoD. For the 10 cases in which the lungs provided crucial information, pathology alone was sufficient. For the four cases in which placenta was a leading factor, pathology alone was sufficient in three cases and in one case, both placental pathology and microbiology were required for CoD assignment.

### Validation of the s-MITS against gold-standard CA (CaDMIA-Plus cohort)

Considering that the data from the lungs and placenta were the most relevant for CoD assignment, the CaDMIA-Plus cases were re-assessed by a different expert panel, that only had access to the laboratory results of the placenta and the lung (modelling the proposed s-MITS). The CoD between the s-MITS model and CA was further compared.

The concordance between the CoD assigned by the modelled s-MITS and those determined by CA in the CaDMIA-Plus study is shown in online supplemental table S1. Complete agreement between s-MITS and CA-based CoD diagnoses was found in 82 out of 90 cases for the main CoD categories. The overall concordance for the CoD category was substantial, with a Kappa value of 0.79 (95% CI 0.65 to 0.92, p<0.001).

The diagnostic performance of s-MITS (considering only the lungs and placenta) compared with the gold standard CA is shown online supplemental table S3. The sensitivity for detecting CA-determined CoD using only lung and placental results ranged from 0.75 to 0.98 across different CoD categories, while the specificity ranged from 0.79 to 1.00.

The complete list of CoD diagnoses for each case in the CaDMIA-Plus cohort, based on gold standard CA results, c-MITS results and results from the lungs and placenta using c-MITS (modelling s-MITS), is provided in online supplemental table S4.

### Comparison between CoD using c-MITS and s-MITS (CaDMIA-Plus vs MIBio cohorts)

**Figure 2:**
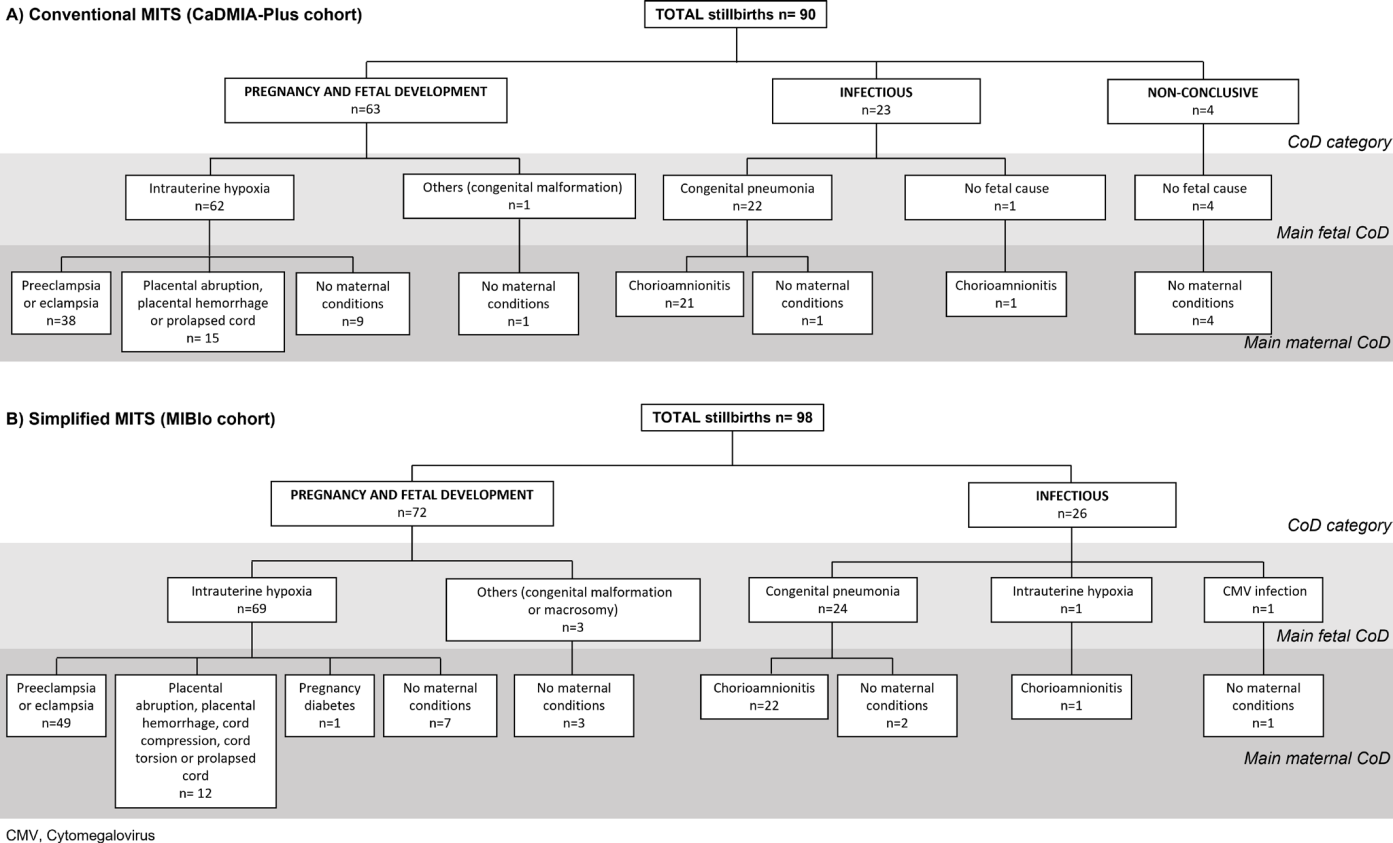
Main cause of death (CoD) category, main fetal CoD and main maternal CoD identified in each case in the CaDMIA-Plus and MIBio cohorts. The diagrams display the total number of cases reported for each general CoD category, as well as the main fetal and maternal CoD in the CaDMIA-Plus cohort (using conventional minimally invasive tissue sampling [c-MITS]) (**A**) and the MIBio cohort (using simplified MITS [s-MITS]) (**B**). CaDMIA-Plus, *Cause of Death investigation using Minimally Invasive Autopsy-Plus;* CMV*, cytomegalovirus;* MIBio*, Mortality Identification Biomarkers.*

**Figure 3:**
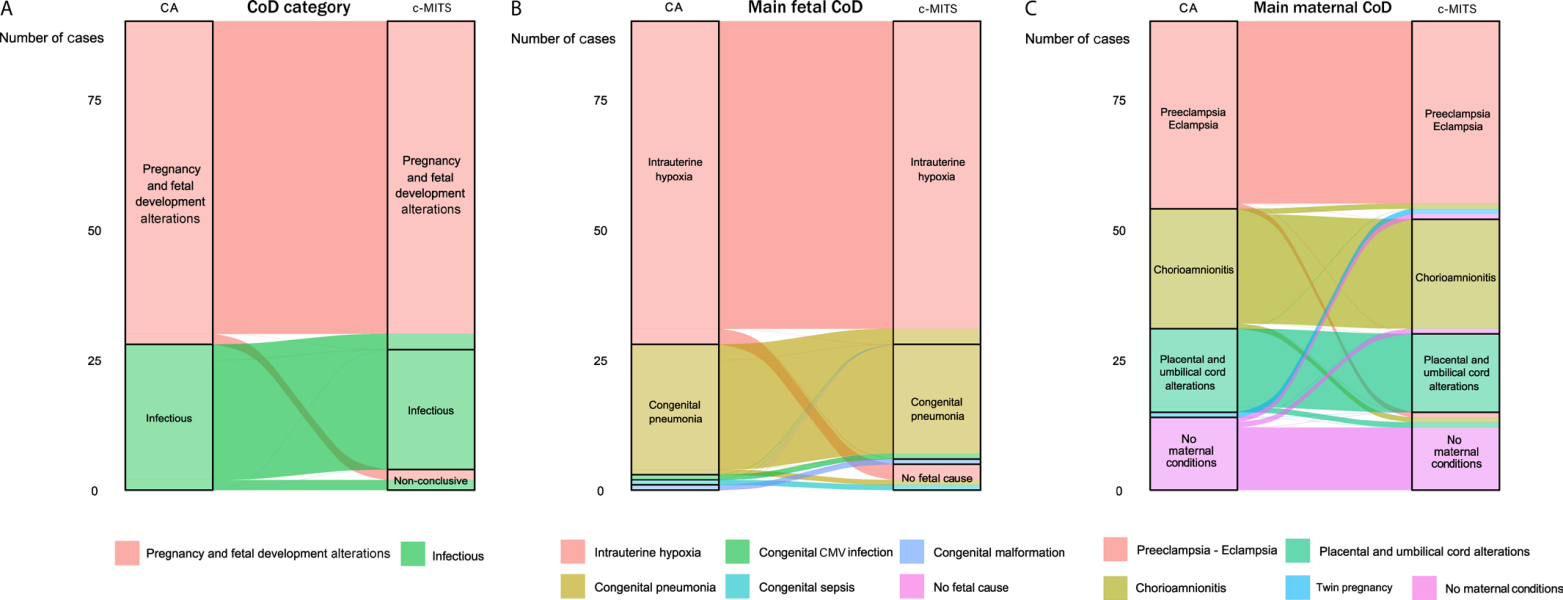
Concordance between the causes of death (CoD) determined using conventional minimally invasive tissue sampling (c-MITS) and the gold standard conventional autopsy (CA) in the CaDMIA-Plus cohort. The alluvial diagrams show the total number of cases assigned to each CoD across the following CoD categories: overall CoD category (**A**), main fetal CoD (**B**) and main maternal CoD (**C**). The stacked blocks represent the CoD determined by CA (left) and by c-MITS (right). The branches between blocks represent differences in the composition of the CoDs between the CA and c-MITS, with their thickness being proportional to the number of cases contained in both blocks connected by the branch. Each CoD is represented by a different colour, which is the same in both diagnostic methods. The colour of the branches is determined by the CA-derived CoD (left). The concordant cases between the CA and c-MITS are represented by branches connected to blocks of the same colour, whereas misclassified cases are shown as branches connected to blocks of a different colour. CaDMIA-Plus, *Cause of Death investigation using Minimally Invasive Autopsy-Plus;* MIBio*, Mortality Identification Biomarkers.*

Table 2 outlines the CoD for the two main cohorts (CaDMIA-Plus and MIBio). The results of the CaDMIA-Plus cohort are shown using both all available information from the c-MITS and using only the information provided by the lungs and placenta (modelled s-MITS), whereas the results of the MIBio cohort include only results based on the s-MITS procedures that were actually performed. No significant differences in the distribution of CoD categories (p=0.139), main fetal CoD (p=0.113) and main maternal CoD (p=0.677) were identified between the CaDMIA-Plus cohort with CoD assigned by c-MITS and the MIBio cohort with CoD assigned by s-MITS. Pregnancy and fetal development alterations were the most common CoD category in both cohorts, followed by infectious diseases and non-conclusive cases. figure 2 summarises the main CoD categories, the main fetal and maternal CoDs identified in the CaDMIA-Plus and MIBio cohorts using c-MITS and s-MITS, and figure 3 illustrates the concordance between CoD determined by these methods.

The complete list of CoD diagnoses for each case in the MIBio cohort, based on c-MITS results is provided in online supplemental table S5.

**Table 2:** Description of causes of death (CoD) for the two main cohorts (CaDMIA-Plus and MIBio). The results of the CaDMIA-Plus cohort are shown in two separate columns: using all information from the performed conventional minimally invasive tissue sampling (MITS) and using only the information provided by the lungs and placenta (modelled simplified MITS). The CoD results of the MIBio cohort include only those based on the performed simplified MITS

	CaDMIA-Plus, n=90			MIBio, n=98	
	Conventional MITS	Modelled simplified MITS	P value*	Simplified MITS	P value* (Conventional MITS vs simplified MITS)
Cause of death category			0.676		0.139
Pregnancy and fetal development	63 (70.0)	67 (74.4)		72 (73.5)	
Infectious	23 (25.6)	21 (23.3)		26 (26.5)	
Non-conclusive	4 (4.4)	2 (2.2)		0 (0.0)	
Main fetal cause of death			0.804		0.113
Intrauterine hypoxia	62 (68.9)	64 (71.1)		70 (71.4)	
Congenital pneumonia	22 (24.4)	23 (25.6)		24 (24.5)	
Congenital cytomegalovirus infection	0 (0.0)	0 (0.0)		1 (1.0)	
Congenital malformation	1 (1.1)	1 (1.1)		2 (2.0)	
Fetal macrosomy	0 (0.0)	0 (0.0)		1 (1.0)	
No fetal cause	5 (5.6)	2 (2.2)		0 (0.0)	
Main maternal cause of death			>0.999		0.677
Pre-eclampsia–eclampsia†	38 (42.2)^‡^	39 (43.3)^‡^		49 (50.0)^§^	
Chorioamnionitis	22 (24.4)	22 (24.4)		23 (23.5)	
Placental/umbilical cord alterations	15 (16.7)	14 (15.6)		12 (12.2)	
Diabetes mellitus in pregnancy	0 (0.0)	0 (0.0)		1 (1.0)	
No maternal conditions	15 (16.7)	15 (16.7)		13 (13.3)	

Categorical variables are summarised as total counts and percentages.

* Fisher’s exact test.

† Pre-eclampsia was defined by hypertension and proteinuria after 20 weeks of gestation. Eclampsia was diagnosed by the clinical evidence of seizures.

‡ 10 women had eclampsia.

§ 11 women had eclampsia.

CaDMIA-Plus, Cause of Death investigation using Minimally Invasive Autopsy-Plus; MIBio, Mortality Identification Biomarkers.; MITS, minimally invasive tissue sampling.

### Time and cost reduction advantage of simplified MITS

On average, the total time required to complete a c-MITS was 85 min: 45 min for performing the procedure (ie, sampling), 20 min for histological evaluation and 20 min for the panel discussion of each case, which involves clinical review and integration of histological and microbiological findings for CoD determination. In contrast, the s-MITS required an average of only 45 min: 25 min for sampling, 10 min for histological evaluation and 10 min for panel discussion, representing a time reduction of approximately 47%.

In addition to significant time efficiency, the s-MITS protocol also demonstrated a clear advantage in terms of cost savings. The estimated total cost, including both the materials needed for sample collection and the subsequent analyses of the c-MITS, which involved extensive laboratory testing of blood, CSF, lungs, liver, CNS and placenta, was €752. In comparison, the total cost of the s-MITS, which only included pathological and microbiological evaluation of the lungs and placenta, was €333. Thus, the s-MITS protocol resulted in a 55.7% reduction of cost compared with c-MITS. A detailed list of the main sample collection tools and the laboratory analysis costs for each sample is provided in table 3.

**Table 3:** Costs in euros (€) of the main materials and analyses included in conventional minimally invasive tissue sampling (c-MITS) and the simplified MITS (s-MITS) protocol.

	Conventional MITS		Simplified MITS	
Type of sample	Evaluation	**Cost (€)**	Evaluation	**Cost (€)**
Blood				
Quincke needle	Yes	0.9	No	0
Microbiology	Yes	67.4	No	0
Cerebrospinal fluid				
Quincke needle	Yes	0.9	No	0
Microbiology	Yes	58.4	No	0
Central nervous system				
Biopsy needle (Biomol)	Yes	27.0	No	0
Microbiology	Yes	80.4	No	0
Pathology	Yes	40.0	No	0
Liver				
Biopsy needle (Bard)	Yes	23.0	No	0
Microbiology	Yes	80.4	No	0
Pathology	Yes	40.0	No	0
Lungs				
Biopsy needle (Bard)	Yes	23.0	Yes	23.0
Microbiology	Yes	80.4	Yes	80.4
Pathology	Yes	40.0	Yes	40.0
Placental disk/blood				
Microbiology	Yes	60.9	Yes	60.9
Pathology	Yes	40.0	Yes	40.0
Umbilical cord/membranes				
Microbiology	Yes	48.9	Yes	48.9
Pathology	Yes	40.0	Yes	40.0
Total cost		**751.6**		**333.2**

MITS, minimally invasive tissue sampling .

## Discussion

In this study, which represents the largest cohort of stillbirths in which MITS has been validated against CA, we confirmed the diagnostic validity of MITS in this age group. Moreover, our findings show that the c-MITS procedure for stillbirths can be further simplified into a cost-effective and time-efficient s-MITS protocol. Importantly, this study helps to address a critical gap in the availability of high-quality CoD data for stillbirths in LMICs by confirming the diagnostic validity and operational feasibility of a s-MITS approach tailored to resource-constrained settings.

The high concordance rates between c-MITS and the gold standard CA for overall CoD category, as well as for both main fetal and maternal CoD, are consistent with previous studies conducted by our group,^15 16^ which showed high concordance rates in stillbirths (Kappa=0.78) compared with other age groups, such as neonates (Kappa=0.40). It should be emphasised, however, that in the previous studies, the MITS results were analysed blindly without any clinical data to determine the validity of the tool by itself.

The analysis of the key data for CoD diagnosis in c-MITS revealed that the analysis of the placenta and lungs was pivotal in determining CoD. In 79.1% of cases, the examination of both organs was necessary for accurate CoD diagnosis, with pathological results alone being sufficient in the majority of these cases. This finding underscores the importance of detailed placental examination in stillbirths, as recommended by the Amsterdam Placental Workshop Consensus.^34^ Also, the significant role of clinical data in diagnosing maternal conditions, such as preeclampsia and eclampsia, highlights the need for collecting comprehensive maternal clinical histories in order to improve diagnostic accuracy. The limited diagnostic contribution of microbiology, especially in the absence of clinical or pathological findings, suggests that the focus in MITS should remain on thorough pathological examination of key organs. Our results corroborate the findings from previous research showing that placental and lung histological findings are very informative, whereas liver and CNS contribute little to CoD determination in stillbirth, while introducing technical difficulties and adding cost to the MITS procedure.^1939^,^41^

A significant milestone of this study was the evaluation of an abbreviated, cost-effective and time-efficient s-MITS protocol, which showed comparable agreement rates with the gold standard CA. The high agreement between CoD determined by only considering the most diagnostically relevant organs (lung and placenta) and those determined by the gold standard CA, with Kappa values ranging from 0.79 to 0.88, highlights the accuracy of this new simplified method. Furthermore, the comparison between two independent stillbirth cohorts using c-MITS (collecting blood, CSF, liver, CNS, lungs and placenta) and s-MITS (collecting only lungs and placenta) revealed no significant differences in the overall prevalence and distribution of CoD between the two cohorts. The 55.7% reduction in cost associated with s-MITS is significant, highlighting the potential of this simplified protocol for broader implementation in LMICs where healthcare budgets are often constrained. Furthermore, in c-MITS, microbiological evaluation contributes to CoD diagnosis in only a minority of cases, suggesting that sample analysis and associated costs could be further reduced. In this regard, future studies could explore stepwise microbiological analysis, where testing is prioritised only when pathological findings suggest infection.

The leading CoD category in the two cohorts evaluated was pregnancy and fetal development alterations (70.0–72.4%), comprising mainly maternal hypertensive disorders and functional placental and/or umbilical cord alterations. The second major CoD was infection-related (25.6–27.6%), consisting mainly of congenital fetal pneumonia secondary to chorioamnionitis (23.5–24.4%). This distribution of CoD is consistent with another report including 984 stillbirths in India and Pakistan,^39^ which also identified pregnancy and fetal development alterations as the major CoD category and a smaller proportion of infection-related deaths. This congruence across different studies suggests that the patterns observed in our cohorts are reflective of broader regional and possibly global trends, at least among LMICs.

While our study provides strong evidence for the diagnostic accuracy and feasibility of MITS and its simplified version (s-MITS) in determining the CoD in stillbirths, certain limitations should be acknowledged. First, the sample size, including 90 cases in the CaDMIA-Plus cohort and 98 cases in the MIBio cohort, along with the single-site design, with all procedures conducted at a single quaternary care centre, may limit the generalisability of our findings. Larger cohorts from geographically diverse LMICs might reveal differences in CoD prevalence, indicating varying diagnostic capabilities between c-MITS and s-MITS that may not have been captured in this study.

Also, it is important to note that comparing the CoD categories between the two cohorts (CaDMIA-Plus using c-MITS and MIBio using s-MITS) shows similarities in CoD patterns but does not confirm the diagnostic accuracy of s-MITS, as different cohorts are characterised. For this specific purpose, we modelled s-MITS within the CaDMIA-Plus cohort by interpreting only the lung and placenta results. However, this approach may introduce some bias, and a direct comparison of the performance of s-MITS and CA within the same population would yield more robust findings.

Additionally, while the exclusion of certain samples in the s-MITS protocol is cost-effective, it may overlook a small percentage of cases in which these samples could have possibly provided critical diagnostic information, such as the liver in cases of metabolic syndromes or congenital syphilis, or CNS/CSF in cases of toxoplasmosis.^42^ Moreover, most fetal malformations cannot be captured by methods that do not include gross examination of organs, such as MITS, either conventional or simplified. These limitations should be taken into account when interpreting our results and underscore the need for further validation studies in diverse settings with larger populations, and its evaluation in cases in which CNS, liver or CSF samples might be more diagnostically relevant. Such studies could help refine and optimise the s-MITS protocol for improved diagnostic precision across various clinical environments. Additionally, the requirement to collect placental tissue for accurate CoD determination, as shown in this study, demands additional effort, as this tissue is not part of the stillbirth and must be obtained separately. While their collection and analysis can enhance diagnostic accuracy, this may pose logistical challenges, especially in clinical settings within LMICs.

Moreover, the cost analysis of c-MITS compared with s-MITS presented in this study includes only direct costs related to materials and laboratory analyses, providing a relative cost estimate between the two procedures under comparable conditions, but does not include labour-related costs, which can vary substantially across different settings. To inform broader implementation decisions, future studies should undertake a more comprehensive cost-effectiveness evaluation that also considers human resources, infrastructure, training and long-term sustainability.

Finally, the broad implementation of MITS in LMICs, in its conventional or simplified form, inevitably requires the proper training of healthcare professionals and addressing cultural barriers to increase acceptability.^43^,^45^ In this regard, initiatives like CHAMPS, which is implementing MITS across seven sites in sub-Saharan Africa and South Asia, along with the appropriate community sensitisation and engagement activities, will be critical for the broad implementation of MITS-based mortality surveillance.^46^

## Conclusion

In summary, this study shows that MITS, including its simplified, cost-effective, and less resource-intensive version, is a reliable alternative to CA for determining CoD in stillbirths, yielding results largely comparable to those of the gold standard CA. By providing robust data on the causes of stillbirth, s-MITS can play a crucial role in informing public health strategies aimed at reducing stillbirth rates globally. The findings also highlight the importance of placental and lung pathology in postmortem evaluations of stillbirths, which should remain central to MITS protocols. Future research should focus on expanding the implementation of s-MITS across diverse settings and continuously refining the technique to further enhance its diagnostic accuracy and cost-effectiveness.

## Supplementary material

10.1136/bmjgh-2024-018380 online supplemental file 1

## Data Availability

All data relevant to the study are included in the article or uploaded as supplementary information. Additional data are available upon request to the corresponding author.
