# Supplementary material for: Diagnostic accuracy of a simplified minimally invasive tissue sampling protocol for stillbirths in low-resource settings
Source: BMJ Glob Health. 2025 Oct 28;10(10):e018380. doi: 10.1136/bmjgh-2024-018380 (PMC12570908; doi:10.1136/bmjgh-2024-018380)
Supplement: online supplemental file 1 [file bmjgh-10-10-s001.docx]

**Supplementary Table 1.** Kappa analysis comparing causes of death (CoD) assigned by conventional minimally invasive tissue sampling (MITS) and simplified MITS to those assigned by the gold-standard (conventional autopsy [CA]) in the CaDMIA-Plus cohort. Interpretation of kappa values follows the criteria of Landis & Koch (1977).

|  | Kappa (95% CI) | Interpretation | Z-value | P-value |
| --- | --- | --- | --- | --- |
| *Conventional MITS- attributed CoD vs. CA-attributed CoD* | | | | |
| CoD category | 0.82 (0.70, 0.94) | Almost perfect | 8.48 | **p<0.001** |
| Fetal CoD | 0.78 (0.66, 0.91) | Substantial | 8.82 | **p<0.001** |
| Maternal CoD | 0.89 (0.81, 0.97) | Almost perfect | 14.18 | **p<0.001** |
| *Simplified MITS-attributed- CoD vs. CA-attributed CoD* | | | | |
| CoD category | 0.79 (0.65, 0.92) | Substantial | 7.88 | **p<0.001** |
| Fetal CoD | 0.85 (0.74, 0.96) | Almost perfect | 9.06 | **p<0.001** |
| Maternal CoD | 0.88 (0.80, 0.96) | Almost perfect | 14.32 | **p<0.001** |

CI; confidence interval

**Supplementary Table 2.** Diagnostic performance measures of conventional minimally invasive tissue sampling (c-MITS) compared to the gold standard of conventional autopsy. Sensitivity, specificity, accuracy, positive and negative predictive values (PPV and NPV), and positive and negative likelihood ratios (LR+ and LR-) along with their 95% confidence intervals (CI), are presented. Cases categorized as cytomegalovirus infection, congenital sepsis, and congenital malformation as main fetal causes of death (CoD) were excluded, as only one case of each was identified by complete autopsy.

|  | | **Sensitivity(**95%CI) | **Specificity(**95%CI) | **Accuracy(**95%CI) | **PPV(**95%CI) | **NPV(**95%CI) | **LR+(**95%CI) | **LR-(**95%CI) |
| --- | --- | --- | --- | --- | --- | --- | --- | --- |
| **CoD category** | Pregnancy and fetal development alterations | 0.97 (0.89, 1) | 0.89 (0.72, 0.98) | 0.94 (0.88, 0.98) | 0.95 (0.87, 0.99) | 0.93 (0.76, 0.99) | 9.03 (3.1, 26.34) | 0.04 (0.01, 0.14) |
|  | Infectious | 0.82 (0.63, 0.94) | 1 (0.94, 1) | 0.94 (0.88, 0.98) | 1 (0.85, 1) | 0.93 (0.83, 0.98) | Inf (NaN, Inf) | 0.18 (0.08, 0.4) |
| **Main fetal CoD** | Congenital pneumonia | 0.84 (0.64, 0.95) | 0.98 (0.92, 1) | 0.94 (0.88, 0.98) | 0.95 (0.77, 1) | 0.94 (0.86, 0.98) | 54.6 (7.75, 384.67) | 0.16 (0.07, 0.4) |
|  | Intrauterine hypoxia | 0.95 (0.87, 0.99) | 0.89 (0.72, 0.98) | 0.93 (0.86, 0.98) | 0.95 (0.87, 0.99) | 0.89 (0.72, 0.98) | 8.88 (3.04, 25.91) | 0.05 (0.02, 0.16) |
| **Main maternal CoD** | Chorioamnionitis | 0.91 (0.72, 0.99) | 0.99 (0.92, 1) | 0.97 (0.91, 0.99) | 0.95 (0.77, 1) | 0.97 (0.9, 1) | 61.17 (8.71, 429.7) | 0.09 (0.02, 0.33) |
|  | Preeclampsia - Eclampsia | 0.97 (0.85, 1) | 0.94 (0.85, 0.99) | 0.96 (0.89, 0.99) | 0.92 (0.79, 0.98) | 0.98 (0.9, 1) | 17.5 (5.82, 52.63) | 0.03 (0, 0.2) |
|  | Placental and umbilical cord alterations | 0.94 (0.7, 1) | 1 (0.95, 1) | 0.99 (0.94, 1) | 1 (0.78, 1) | 0.99 (0.93, 1) | Inf (NaN, Inf) | 0.06 (0.01, 0.42) |
|  | No maternal conditions | 0.86 (0.57, 0.98) | 0.96 (0.89, 0.99) | 0.94 (0.88, 0.98) | 0.8 (0.52, 0.96) | 0.97 (0.91, 1) | 21.71 (7.02, 67.18) | 0.15 (0.04, 0.54) |

Inf: infinite; NaN: not a number, insufficient observations.

**Supplementary Table 3.** Diagnostic performance measures of the simplified-minimally invasive tissue sampling compared to the gold standard of conventional autopsy. Sensitivity, specificity, accuracy, positive and negative predictive values (PPV and NPV), and positive and negative likelihood ratios (LR+ and LR-), along with their 95% confidence intervals (CI), are presented. Categories with one or zero cases were excluded from the analysis, including the “non-conclusive” cause of death (CoD) category, as no non-conclusive cases were identified by conventional autopsy in this cohort. Additionally, cases categorized as cytomegalovirus infection, congenital sepsis, and congenital malformation as main fetal CoD were excluded, as only one case of each was identified by conventional autopsy.

|  | | **Sensitivity(**95%CI) | **Specificity(**95%CI) | **Accuracy(**95%CI) | **PPV (**95%CI) | **NPV (**95%CI) | **LR+ (**95%CI) | **LR- (**95%CI) |
| --- | --- | --- | --- | --- | --- | --- | --- | --- |
| **CoD category** | Pregnancy and fetal development alterations | 0.98 (0.91, 1) | 0.79 (0.59, 0.92) | 0.92 (0.85, 0.97) | 0.91 (0.82, 0.97) | 0.96 (0.78, 1) | 4.59 (2.26, 9.34) | 0.02 (0, 0.14) |
|  | Infectious | 0.75 (0.55, 0.89) | 1 (0.94, 1) | 0.92 (0.85, 0.97) | 1 (0.84, 1) | 0.9 (0.8, 0.96) | Inf (NaN, Inf) | 0.25 (0.13, 0.47) |
| **Main fetal CoD** | Congenital pneumonia | 0.88 (0.69, 0.97) | 0.98 (0.92, 1) | 0.96 (0.89, 0.99) | 0.96 (0.78, 1) | 0.96 (0.87, 0.99) | 57.2 (8.14, 402.12) | 0.12 (0.04, 0.35) |
|  | Intrauterine hypoxia | 0.98 (0.91, 1) | 0.89 (0.72, 0.98) | 0.96 (0.89, 0.99) | 0.95 (0.87, 0.99) | 0.96 (0.8, 1) | 9.18 (3.15, 26.76) | 0.02 (0, 0.13) |
| **Main maternal CoD** | Chorioamnionitis | 0.91 (0.72, 0.99) | 0.99 (0.92, 1) | 0.97 (0.91, 0.99) | 0.95 (0.77, 1) | 0.97 (0.9, 1) | 61.17 (8.71, 429.7) | 0.09 (0.02, 0.33) |
|  | Preeclampsia – Eclampsia | 0.97 (0.85, 1) | 0.93 (0.82, 0.98) | 0.94 (0.88, 0.98) | 0.9 (0.76, 0.97) | 0.98 (0.9, 1) | 13.12 (5.1, 33.75) | 0.03 (0, 0.21) |
|  | Placental and umbilical cord alterations | 0.88 (0.62, 0.98) | 1 (0.95, 1) | 0.98 (0.92, 1) | 1 (0.77, 1) | 0.97 (0.91, 1) | Inf (NaN, Inf) | 0.12 (0.03, 0.46) |
|  | No maternal conditions | 0.86 (0.57, 0.98) | 0.96 (0.89, 0.99) | 0.94 (0.88, 0.98) | 0.8 (0.52, 0.96) | 0.97 (0.91, 1) | 21.71 (7.02, 67.18) | 0.15 (0.04, 0.54) |

Inf: infinite; NaN: not a number, insufficient observations.

**Supplementary Table 4.** List of causes of death (CoD) assigned to all cases in the CaDMIA-Plus cohort, including CoD category, main fetal and maternal CoD assigned by the gold-standard conventional autopsy, by conventional minimally invasive tissue sampling using all organ results, and by simplified MITS considering only lung and placenta results. Additionally, the table includes fetal gestational age and specifies the main maternal and/or fetal infectious agents for cases categorized as infectious CoD.

|  | | **Causes of death (CoD) and microbiological results captured by gold-standard conventional autopsy** | | | **Causes of death (CoD) and microbiological results captured by conventional minimally invasive tissue sampling** | | | **Causes of death (CoD) and microbiological results that would have been captured by a simplified minimally invasive tissue sampling** | | |
| --- | --- | --- | --- | --- | --- | --- | --- | --- | --- | --- |
| Study Number | **Gestational age (weeks)** | **CoD category** | **Main fetal CoD** | **Main maternal CoD** | **CoD group** | **Main fetal CoD** | **Main maternal CoD** | **CoD category** | **Main fetal CoD** | **Main maternal CoD** |
| 1 | ND | Infectious | Congenital pneumonia (No agent) | Chorioamnionitis (No agent) | Infectious | Congenital pneumonia (No agent) | Chorioamnionitis (No agent) | Infectious | Congenital pneumonia (No agent) | Chorioamnionitis (No agent) |
| 2 | 38 | Infectious | Congenital pneumonia (Escherichia coli) | No maternal conditions | Infectious | Congenital pneumonia (Escherichia coli) | No maternal conditions | Infectious | Congenital pneumonia (Escherichia coli) | No maternal conditions |
| 3 | 40 | Infectious | Congenital pneumonia (S. agalactiae) | Chorioamnionitis (S. agalactiae) | Infectious | Congenital pneumonia (S. agalactiae) | Chorioamnionitis (S. agalactiae) | Infectious | Congenital pneumonia (S. agalactiae) | Chorioamnionitis (S. agalactiae) |
| 4 | ND | Infectious | Congenital pneumonia (No agent) | Chorioamnionitis (S. agalactiae) | Infectious | Congenital pneumonia (No agent) | Chorioamnionitis (S. agalactiae) | Infectious | Congenital pneumonia (No agent) | Chorioamnionitis (S. agalactiae) |
| 5 | ND | Infectious | Congenital pneumonia (No agent) | Chorioamnionitis (No agent) | Infectious | Congenital pneumonia (No agent) | Chorioamnionitis (No agent) | Infectious | Congenital pneumonia (No agent) | Chorioamnionitis (No agent) |
| 6 | 35 | Infectious | Congenital pneumonia (No agent) | Chorioamnionitis (No agent) | Infectious | Congenital pneumonia (No agent) | Chorioamnionitis (No agent) | Infectious | Congenital pneumonia (No agent) | Chorioamnionitis (No agent) |
| 7 | 39 | Infectious | Congenital pneumonia (No agent) | Chorioamnionitis (No agent) | Infectious | Congenital pneumonia (No agent) | Chorioamnionitis (No agent) | Infectious | Congenital pneumonia (No agent) | Chorioamnionitis (No agent) |
| 8 | ND | Infectious | Congenital pneumonia (No agent) | Chorioamnionitis (No agent) | Infectious | Congenital pneumonia (S. agalactiae) | Chorioamnionitis (S. agalactiae) | Infectious | Congenital pneumonia (S. agalactiae) | Chorioamnionitis (S. agalactiae) |
| 9 | 36 | Infectious | Congenital pneumonia (No agent) | Chorioamnionitis (No agent) | Infectious | Congenital pneumonia (No agent) | Chorioamnionitis (No agent) | Infectious | Congenital pneumonia (No agent) | Chorioamnionitis (No agent) |
| 10 | ND | Infectious | Congenital pneumonia (No agent) | Chorioamnionitis (No agent) | Infectious | Congenital pneumonia (No agent) | Chorioamnionitis (No agent) | Infectious | Congenital pneumonia (No agent) | Chorioamnionitis (No agent) |
| 11 | ND | Infectious | Congenital pneumonia (S. agalactiae) | Chorioamnionitis (S. agalactiae) | Infectious | Congenital pneumonia (S. agalactiae) | Chorioamnionitis (S. agalactiae) | Infectious | Congenital pneumonia (S. agalactiae) | Chorioamnionitis (S. agalactiae) |
| 12 | 31 | Infectious | Congenital cytomegalovirus infection | No maternal conditions | Infectious | Congenital pneumonia (No agent) | Chorioamnionitis (No agent) | Infectious | Congenital pneumonia (No agent) | Chorioamnionitis (No agent) |
| 13 | 40 | Infectious | Congenital pneumonia (No agent) | Chorioamnionitis (No agent) | Infectious | Congenital pneumonia (No agent) | Chorioamnionitis (No agent) | Infectious | Congenital pneumonia (No agent) | Chorioamnionitis (No agent) |
| 14 | 32 | Infectious | Congenital pneumonia (No agent) | Chorioamnionitis (S. agalactiae) | Infectious | Congenital pneumonia (S. agalactiae) | Chorioamnionitis (S. agalactiae) | Infectious | Congenital pneumonia (S. agalactiae) | Chorioamnionitis (S. agalactiae) |
| 15 | ND | Infectious | Congenital pneumonia (S. agalactiae) | Chorioamnionitis (S. agalactiae) | Infectious | Congenital pneumonia (S. agalactiae) | Chorioamnionitis (S. agalactiae) | Infectious | Congenital pneumonia (S. agalactiae) | Chorioamnionitis (S. agalactiae) |
| 16 | 40 | Infectious | Congenital pneumonia (No agent) | Chorioamnionitis (S. agalactiae) | Infectious | Congenital pneumonia (S. agalactiae) | Chorioamnionitis (S. agalactiae) | Infectious | Congenital pneumonia (S. agalactiae) | Chorioamnionitis (S. agalactiae) |
| 17 | ND | Infectious | Congenital pneumonia (No agent) | Chorioamnionitis (No agent) | Infectious | Congenital pneumonia (No agent) | Chorioamnionitis (No agent) | Infectious | Congenital pneumonia (No agent) | Chorioamnionitis (No agent) |
| 18 | 31 | Infectious | Congenital pneumonia (No agent) | Chorioamnionitis (No agent) | Infectious | Congenital pneumonia (No agent) | Chorioamnionitis (No agent) | Infectious | Congenital pneumonia (No agent) | Chorioamnionitis (No agent) |
| 19 | 38 | Infectious | Congenital pneumonia (No agent) | Chorioamnionitis (No agent) | Infectious | Congenital pneumonia (No agent) | Chorioamnionitis (No agent) | Infectious | Congenital pneumonia (No agent) | Chorioamnionitis (No agent) |
| 20 | ND | Infectious | Congenital pneumonia (No agent) | Chorioamnionitis (No agent) | Infectious | Congenital pneumonia (No agent) | Chorioamnionitis (No agent) | Infectious | Congenital pneumonia (No agent) | Chorioamnionitis (No agent) |
| 21 | ND | Infectious | Congenital pneumonia (No agent) | Chorioamnionitis (No agent) | Infectious | Congenital pneumonia (No agent) | Chorioamnionitis (No agent) | Infectious | Congenital pneumonia (No agent) | Chorioamnionitis (No agent) |
| 22 | 32 | Infectious | Congenital pneumonia (No agent) | Chorioamnionitis (S. agalactiae) | Infectious | Congenital pneumonia (S. agalactiae) | Chorioamnionitis (S. agalactiae) | Pregnancy and fetal development | Congenital pneumonia (S. agalactiae) | Chorioamnionitis (S. agalactiae) |
| 23 | 36 | Infectious | Congenital pneumonia (S. agalactiae) | Chorioamnionitis (S. agalactiae) | Infectious | No fetal cause | Chorioamnionitis (S. agalactiae) | Pregnancy and fetal development | Intrauterine hypoxia | Preeclampsia |
| 24 | 34 | Infectious | Intrauterine hypoxia | Chorioamnionitis (S. agalactiae) | Non-conclusive | No fetal cause | No maternal conditions | Pregnancy and fetal development | Intrauterine hypoxia | Preeclampsia |
| 25 | 39 | Infectious | Congenital sepsis (S. agalactiae) | No maternal conditions | Non-conclusive | No fetal cause | No maternal conditions | Pregnancy and fetal development | Intrauterine hypoxia | No maternal conditions |
| 26 | 39 | Infectious | Congenital pneumonia (S. agalactiae) | No maternal conditions | Pregnancy and fetal development | Intrauterine hypoxia | No maternal conditions | Non-conclusive | No fetal cause | No maternal conditions |
| 27 | 34 | Infectious | Congenital pneumonia (S. agalactiae) | No maternal conditions | Pregnancy and fetal development | Intrauterine hypoxia | No maternal conditions | Pregnancy and fetal development | Intrauterine hypoxia | No maternal conditions |
| 28 | 33 | Infectious | Congenital pneumonia (No agent) | Chorioamnionitis (No agent) | Pregnancy and fetal development | Intrauterine hypoxia | Preeclampsia - Eclampsia | Pregnancy and fetal development | Congenital pneumonia (No agent) | Preeclampsia - Eclampsia |
| 29 | 39 | Pregnancy and fetal development | Intrauterine hypoxia | No maternal conditions | Non-conclusive | No fetal cause | No maternal conditions | Non-conclusive | No fetal cause | No maternal conditions |
| 30 | ND | Pregnancy and fetal development | Intrauterine hypoxia | No maternal conditions | Non-conclusive | No fetal cause | No maternal conditions | Pregnancy and fetal development | Intrauterine hypoxia | No maternal conditions |
| 31 | 30 | Pregnancy and fetal development | Intrauterine hypoxia | Twin pregnancy | Pregnancy and fetal development | Intrauterine hypoxia | Preeclampsia - Eclampsia | Pregnancy and fetal development | Intrauterine hypoxia | Preeclampsia - Eclampsia |
| 32 | 29 | Pregnancy and fetal development | Intrauterine hypoxia | Preeclampsia - Eclampsia | Pregnancy and fetal development | Intrauterine hypoxia | Preeclampsia - Eclampsia | Pregnancy and fetal development | Intrauterine hypoxia | Preeclampsia - Eclampsia |
| 33 | 31 | Pregnancy and fetal development | Intrauterine hypoxia | Preeclampsia - Eclampsia | Pregnancy and fetal development | Intrauterine hypoxia | Preeclampsia - Eclampsia | Pregnancy and fetal development | Intrauterine hypoxia | Preeclampsia - Eclampsia |
| 34 | 30 | Pregnancy and fetal development | Intrauterine hypoxia | Preeclampsia - Eclampsia | Pregnancy and fetal development | Intrauterine hypoxia | Preeclampsia - Eclampsia | Pregnancy and fetal development | Intrauterine hypoxia | Preeclampsia - Eclampsia |
| 35 | 34 | Pregnancy and fetal development | Intrauterine hypoxia | Preeclampsia - Eclampsia | Pregnancy and fetal development | Intrauterine hypoxia | Preeclampsia - Eclampsia | Pregnancy and fetal development | Intrauterine hypoxia | Preeclampsia - Eclampsia |
| 36 | 35 | Pregnancy and fetal development | Intrauterine hypoxia | Preeclampsia - Eclampsia | Pregnancy and fetal development | Intrauterine hypoxia | Preeclampsia - Eclampsia | Pregnancy and fetal development | Intrauterine hypoxia | Preeclampsia - Eclampsia |
| 37 | 34 | Pregnancy and fetal development | Intrauterine hypoxia | Preeclampsia - Eclampsia | Pregnancy and fetal development | Intrauterine hypoxia | Preeclampsia - Eclampsia | Pregnancy and fetal development | Intrauterine hypoxia | Preeclampsia - Eclampsia |
| 38 | 37 | Pregnancy and fetal development | Intrauterine hypoxia | Preeclampsia - Eclampsia | Pregnancy and fetal development | Intrauterine hypoxia | Preeclampsia - Eclampsia | Pregnancy and fetal development | Intrauterine hypoxia | Preeclampsia - Eclampsia |
| 39 | 34 | Pregnancy and fetal development | Intrauterine hypoxia | Placental and umbilical cord alterations | Pregnancy and fetal development | Intrauterine hypoxia | Placental and umbilical cord alterations | Pregnancy and fetal development | Intrauterine hypoxia | Placental and umbilical cord alterations |
| 40 | 38 | Pregnancy and fetal development | Intrauterine hypoxia | Preeclampsia - Eclampsia | Pregnancy and fetal development | Intrauterine hypoxia | Preeclampsia - Eclampsia | Pregnancy and fetal development | Intrauterine hypoxia | Preeclampsia - Eclampsia |
| 41 | 33 | Pregnancy and fetal development | Intrauterine hypoxia | Preeclampsia - Eclampsia | Pregnancy and fetal development | Intrauterine hypoxia | Preeclampsia - Eclampsia | Pregnancy and fetal development | Intrauterine hypoxia | Preeclampsia - Eclampsia |
| 42 | 38 | Pregnancy and fetal development | Intrauterine hypoxia | Preeclampsia - Eclampsia | Pregnancy and fetal development | Intrauterine hypoxia | Preeclampsia - Eclampsia | Pregnancy and fetal development | Intrauterine hypoxia | Preeclampsia - Eclampsia |
| 43 | 38 | Pregnancy and fetal development | Intrauterine hypoxia | Placental and umbilical cord alterations | Pregnancy and fetal development | Intrauterine hypoxia | Placental and umbilical cord alterations | Pregnancy and fetal development | Intrauterine hypoxia | Placental and umbilical cord alterations |
| 44 | ND | Pregnancy and fetal development | Intrauterine hypoxia | No maternal conditions | Pregnancy and fetal development | Intrauterine hypoxia | Preeclampsia - Eclampsia | Pregnancy and fetal development | Intrauterine hypoxia | Preeclampsia - Eclampsia |
| 45 | ND | Pregnancy and fetal development | Intrauterine hypoxia | Preeclampsia - Eclampsia | Pregnancy and fetal development | Intrauterine hypoxia | Preeclampsia - Eclampsia | Pregnancy and fetal development | Intrauterine hypoxia | Preeclampsia - Eclampsia |
| 46 | 27 | Pregnancy and fetal development | Intrauterine hypoxia | Preeclampsia - Eclampsia | Pregnancy and fetal development | Intrauterine hypoxia | Preeclampsia - Eclampsia | Pregnancy and fetal development | Intrauterine hypoxia | Preeclampsia - Eclampsia |
| 47 | 37 | Pregnancy and fetal development | Intrauterine hypoxia | Preeclampsia - Eclampsia | Pregnancy and fetal development | Intrauterine hypoxia | Preeclampsia - Eclampsia | Pregnancy and fetal development | Intrauterine hypoxia | Preeclampsia - Eclampsia |
| 48 | 38 | Pregnancy and fetal development | Intrauterine hypoxia | Placental and umbilical cord alterations | Pregnancy and fetal development | Intrauterine hypoxia | Placental and umbilical cord alterations | Pregnancy and fetal development | Intrauterine hypoxia | Placental and umbilical cord alterations |
| 49 | 30 | Pregnancy and fetal development | Intrauterine hypoxia | Preeclampsia - Eclampsia | Pregnancy and fetal development | Intrauterine hypoxia | Preeclampsia - Eclampsia | Pregnancy and fetal development | Intrauterine hypoxia | Preeclampsia - Eclampsia |
| 50 | 23 | Pregnancy and fetal development | Intrauterine hypoxia | Preeclampsia - Eclampsia | Pregnancy and fetal development | Intrauterine hypoxia | Preeclampsia - Eclampsia | Pregnancy and fetal development | Intrauterine hypoxia | Preeclampsia - Eclampsia |
| 51 | ND | Pregnancy and fetal development | Intrauterine hypoxia | Preeclampsia - Eclampsia | Pregnancy and fetal development | Intrauterine hypoxia | Preeclampsia - Eclampsia | Pregnancy and fetal development | Intrauterine hypoxia | Preeclampsia - Eclampsia |
| 52 | ND | Pregnancy and fetal development | Intrauterine hypoxia | No maternal conditions | Pregnancy and fetal development | Intrauterine hypoxia | No maternal conditions | Pregnancy and fetal development | Intrauterine hypoxia | No maternal conditions |
| 53 | ND | Pregnancy and fetal development | Intrauterine hypoxia | Placental and umbilical cord alterations | Pregnancy and fetal development | Intrauterine hypoxia | No maternal conditions | Pregnancy and fetal development | Intrauterine hypoxia | Placental abruption |
| 54 | 31 | Pregnancy and fetal development | Intrauterine hypoxia | Preeclampsia - Eclampsia | Pregnancy and fetal development | Intrauterine hypoxia | Preeclampsia - Eclampsia | Pregnancy and fetal development | Intrauterine hypoxia | Preeclampsia - Eclampsia |
| 55 | 39 | Pregnancy and fetal development | Intrauterine hypoxia | Placental and umbilical cord alterations | Pregnancy and fetal development | Intrauterine hypoxia | Placental and umbilical cord alterations | Pregnancy and fetal development | Intrauterine hypoxia | Placental and umbilical cord alterations |
| 56 | 38 | Pregnancy and fetal development | Congenital malformation | No maternal conditions | Pregnancy and fetal development | Congenital malformation | No maternal conditions | Pregnancy and fetal development | Congenital malformation | No maternal conditions |
| 57 | 32 | Pregnancy and fetal development | Intrauterine hypoxia | Placental and umbilical cord alterations | Pregnancy and fetal development | Intrauterine hypoxia | Placental and umbilical cord alterations | Pregnancy and fetal development | Intrauterine hypoxia | Placental and umbilical cord alterations |
| 58 | ND | Pregnancy and fetal development | Intrauterine hypoxia | Placental and umbilical cord alterations | Pregnancy and fetal development | Intrauterine hypoxia | Placental and umbilical cord alterations | Pregnancy and fetal development | Intrauterine hypoxia | Placental and umbilical cord alterations |
| 59 | 33 | Pregnancy and fetal development | Intrauterine hypoxia | Placental and umbilical cord alterations | Pregnancy and fetal development | Intrauterine hypoxia | Placental and umbilical cord alterations | Pregnancy and fetal development | Intrauterine hypoxia | Placental and umbilical cord alterations |
| 60 | ND | Pregnancy and fetal development | Intrauterine hypoxia | Placental and umbilical cord alterations | Pregnancy and fetal development | Intrauterine hypoxia | Placental and umbilical cord alterations | Pregnancy and fetal development | Intrauterine hypoxia | Placental and umbilical cord alterations |
| 61 | ND | Pregnancy and fetal development | Intrauterine hypoxia | No maternal conditions | Pregnancy and fetal development | Intrauterine hypoxia | No maternal conditions | Pregnancy and fetal development | Intrauterine hypoxia | No maternal conditions |
| 62 | 29 | Pregnancy and fetal development | Intrauterine hypoxia | No maternal conditions | Pregnancy and fetal development | Intrauterine hypoxia | No maternal conditions | Pregnancy and fetal development | Intrauterine hypoxia | No maternal conditions |
| 63 | 40 | Pregnancy and fetal development | Intrauterine hypoxia | Preeclampsia - Eclampsia | Pregnancy and fetal development | Intrauterine hypoxia | Preeclampsia - Eclampsia | Pregnancy and fetal development | Intrauterine hypoxia | Preeclampsia - Eclampsia |
| 64 | 30 | Pregnancy and fetal development | Intrauterine hypoxia | Preeclampsia - Eclampsia | Pregnancy and fetal development | Intrauterine hypoxia | Preeclampsia - Eclampsia | Pregnancy and fetal development | Intrauterine hypoxia | Preeclampsia - Eclampsia |
| 65 | ND | Pregnancy and fetal development | Intrauterine hypoxia | Preeclampsia - Eclampsia | Pregnancy and fetal development | Intrauterine hypoxia | Preeclampsia - Eclampsia | Pregnancy and fetal development | Intrauterine hypoxia | Preeclampsia - Eclampsia |
| 66 | ND | Pregnancy and fetal development | Intrauterine hypoxia | Placental and umbilical cord alterations | Pregnancy and fetal development | Intrauterine hypoxia | Placental and umbilical cord alterations | Pregnancy and fetal development | Intrauterine hypoxia | Placental and umbilical cord alterations |
| 67 | 29 | Pregnancy and fetal development | Intrauterine hypoxia | No maternal conditions | Pregnancy and fetal development | Intrauterine hypoxia | No maternal conditions | Pregnancy and fetal development | Intrauterine hypoxia | No maternal conditions |
| 68 | 38 | Pregnancy and fetal development | Intrauterine hypoxia | Placental and umbilical cord alterations | Pregnancy and fetal development | Intrauterine hypoxia | Placental and umbilical cord alterations | Pregnancy and fetal development | Intrauterine hypoxia | Placental and umbilical cord alterations |
| 69 | 40 | Pregnancy and fetal development | Intrauterine hypoxia | Placental and umbilical cord alterations | Pregnancy and fetal development | Intrauterine hypoxia | Placental and umbilical cord alterations | Pregnancy and fetal development | Intrauterine hypoxia | Placental and umbilical cord alterations |
| 70 | 32 | Pregnancy and fetal development | Intrauterine hypoxia | Preeclampsia - Eclampsia | Pregnancy and fetal development | Intrauterine hypoxia | Preeclampsia - Eclampsia | Pregnancy and fetal development | Intrauterine hypoxia | Preeclampsia - Eclampsia |
| 71 | 28 | Pregnancy and fetal development | Intrauterine hypoxia | Preeclampsia - Eclampsia | Pregnancy and fetal development | Intrauterine hypoxia | Preeclampsia - Eclampsia | Pregnancy and fetal development | Intrauterine hypoxia | Preeclampsia - Eclampsia |
| 72 | ND | Pregnancy and fetal development | Intrauterine hypoxia | Preeclampsia - Eclampsia | Pregnancy and fetal development | Intrauterine hypoxia | Preeclampsia - Eclampsia | Pregnancy and fetal development | Intrauterine hypoxia | Preeclampsia - Eclampsia |
| 73 | ND | Pregnancy and fetal development | Intrauterine hypoxia | Placental and umbilical cord alterations | Pregnancy and fetal development | Intrauterine hypoxia | Placental and umbilical cord alterations | Pregnancy and fetal development | Intrauterine hypoxia | Placental and umbilical cord alterations |
| 74 | 41 | Pregnancy and fetal development | Intrauterine hypoxia | Placental and umbilical cord alterations | Pregnancy and fetal development | Intrauterine hypoxia | Placental and umbilical cord alterations | Pregnancy and fetal development | Intrauterine hypoxia | Placental and umbilical cord alterations |
| 75 | 29 | Pregnancy and fetal development | Intrauterine hypoxia | Preeclampsia - Eclampsia | Pregnancy and fetal development | Intrauterine hypoxia | Preeclampsia - Eclampsia | Pregnancy and fetal development | Intrauterine hypoxia | Preeclampsia - Eclampsia |
| 76 | 30 | Pregnancy and fetal development | Intrauterine hypoxia | Preeclampsia - Eclampsia | Pregnancy and fetal development | Intrauterine hypoxia | Preeclampsia - Eclampsia | Pregnancy and fetal development | Intrauterine hypoxia | Preeclampsia - Eclampsia |
| 77 | 28 | Pregnancy and fetal development | Intrauterine hypoxia | Preeclampsia - Eclampsia | Pregnancy and fetal development | Intrauterine hypoxia | Preeclampsia - Eclampsia | Pregnancy and fetal development | Intrauterine hypoxia | Preeclampsia - Eclampsia |
| 78 | 40 | Pregnancy and fetal development | Intrauterine hypoxia | Placental and umbilical cord alterations | Pregnancy and fetal development | Intrauterine hypoxia | Placental and umbilical cord alterations | Pregnancy and fetal development | Intrauterine hypoxia | Placental and umbilical cord alterations |
| 79 | ND | Pregnancy and fetal development | Intrauterine hypoxia | Preeclampsia - Eclampsia | Pregnancy and fetal development | Intrauterine hypoxia | No maternal conditions | Pregnancy and fetal development | Intrauterine hypoxia | No maternal conditions |
| 80 | 36 | Pregnancy and fetal development | Intrauterine hypoxia | Preeclampsia - Eclampsia | Pregnancy and fetal development | Intrauterine hypoxia | Preeclampsia - Eclampsia | Pregnancy and fetal development | Intrauterine hypoxia | Preeclampsia - Eclampsia |
| 81 | ND | Pregnancy and fetal development | Intrauterine hypoxia | Preeclampsia - Eclampsia | Pregnancy and fetal development | Intrauterine hypoxia | Preeclampsia - Eclampsia | Pregnancy and fetal development | Intrauterine hypoxia | Preeclampsia - Eclampsia |
| 82 | 29 | Pregnancy and fetal development | Intrauterine hypoxia | Preeclampsia - Eclampsia | Pregnancy and fetal development | Intrauterine hypoxia | Preeclampsia - Eclampsia | Pregnancy and fetal development | Intrauterine hypoxia | Preeclampsia - Eclampsia |
| 83 | ND | Pregnancy and fetal development | Intrauterine hypoxia | Preeclampsia - Eclampsia | Pregnancy and fetal development | Intrauterine hypoxia | Preeclampsia - Eclampsia | Pregnancy and fetal development | Intrauterine hypoxia | Preeclampsia - Eclampsia |
| 84 | 32 | Pregnancy and fetal development | Intrauterine hypoxia | No maternal conditions | Pregnancy and fetal development | Intrauterine hypoxia | No maternal conditions | Pregnancy and fetal development | Intrauterine hypoxia | No maternal conditions |
| 85 | 31 | Pregnancy and fetal development | Intrauterine hypoxia | Placental and umbilical cord alterations | Pregnancy and fetal development | Intrauterine hypoxia | Placental and umbilical cord alterations | Pregnancy and fetal development | Intrauterine hypoxia | Placental and umbilical cord alterations |
| 86 | ND | Pregnancy and fetal development | Intrauterine hypoxia | Preeclampsia - Eclampsia | Pregnancy and fetal development | Intrauterine hypoxia | Preeclampsia - Eclampsia | Pregnancy and fetal development | Intrauterine hypoxia | Preeclampsia - Eclampsia |
| 87 | 36 | Pregnancy and fetal development | Intrauterine hypoxia | Preeclampsia - Eclampsia | Pregnancy and fetal development | Intrauterine hypoxia | Preeclampsia - Eclampsia | Pregnancy and fetal development | Intrauterine hypoxia | Preeclampsia - Eclampsia |
| 88 | ND | Pregnancy and fetal development | Intrauterine hypoxia | Preeclampsia - Eclampsia | Pregnancy and fetal development | Intrauterine hypoxia | Preeclampsia - Eclampsia | Pregnancy and fetal development | Intrauterine hypoxia | Preeclampsia - Eclampsia |
| 89 | ND | Pregnancy and fetal development | Intrauterine hypoxia | Preeclampsia - Eclampsia | Pregnancy and fetal development | Intrauterine hypoxia | Preeclampsia - Eclampsia | Pregnancy and fetal development | Intrauterine hypoxia | Preeclampsia - Eclampsia |
| 90 | ND | Pregnancy and fetal development | Intrauterine hypoxia | Preeclampsia - Eclampsia | Pregnancy and fetal development | Intrauterine hypoxia | Preeclampsia - Eclampsia | Pregnancy and fetal development | Intrauterine hypoxia | Preeclampsia - Eclampsia |

ND: Not determined

**Supplementary Table 5.** List of causes of death (CoD) assigned to all cases in the MIBio cohort based on results from the simplified MITS (s-MITS), including CoD category, main fetal and maternal CoD. Additionally, the table includes fetal gestational age and specifies the main maternal and/or fetal infectious agents for cases categorized as infectious CoD.

|  | | **Causes of death (CoD) and microbiological results captured by simplified minimally invasive tissue sampling** | | |
| --- | --- | --- | --- | --- |
| **Study Number** | **Gestational Age (weeks)** | **CoD Category** | **Main maternal CoD** | **Main fetal CoD** |
| 1 | 36 | Infectious | Chorioamnionitis (Mycoplasma) | Congenital pneumonia (Mycoplasma) |
| 2 | 27 | Infectious | No maternal conditions | Congenital pneumonia (S. Agalactiae) |
| 3 | 38.8 | Infectious | Chorioamnionitis (No agent) | Congenital pneumonia (No agent) |
| 4 | 32.3 | Infectious | No maternal conditions | Congenital pneumonia (No agent) |
| 5 | 36.4 | Infectious | Chorioamnionitis (S. vaginalis) | Congenital pneumonia (No agent) |
| 6 | 35 | Infectious | Chorioamnionitis (No agent) | Congenital pneumonia (S. Agalactiae) |
| 7 | 36.4 | Infectious | Chorioamnionitis (No agent) | Congenital pneumonia (No agent) |
| 8 | 37.4 | Infectious | Chorioamnionitis (S. agalactiae) | Congenital pneumonia (S. Agalactiae) |
| 9 | 40.2 | Infectious | Chorioamnionitis (No agent) | Congenital pneumonia (No agent) |
| 10 | 37.6 | Infectious | Chorioamnionitis (No agent) | Congenital pneumonia (No agent) |
| 11 | 41.2 | Infectious | Chorioamnionitis (S. agalactiae) | Congenital pneumonia (S. Agalactiae) |
| 12 | 38.5 | Infectious | Chorioamnionitis (No agent) | Congenital pneumonia (No agent) |
| 13 | 41 | Infectious | Chorioamnionitis (No agent) | Congenital pneumonia (No agent) |
| 14 | 39.5 | Infectious | Chorioamnionitis (No agent) | Congenital pneumonia (No agent) |
| 15 | 31 | Infectious | Chorioamnionitis (S. agalactiae) | Congenital pneumonia (S. Agalactiae) |
| 16 | 31.1 | Infectious | Chorioamnionitis (S. agalactiae) | Congenital pneumonia (S. Agalactiae) |
| 17 | 29.1 | Infectious | Chorioamnionitis (S. agalactiae) | Congenital pneumonia (S. Agalactiae) |
| 18 | 37.3 | Infectious | Chorioamnionitis (No agent) | Congenital pneumonia (No agent) |
| 19 | 40.5 | Infectious | Chorioamnionitis (No agent) | Congenital pneumonia (No agent) |
| 20 | 36.4 | Infectious | Chorioamnionitis (No agent) | Congenital pneumonia (No agent) |
| 21 | 34 | Infectious | No maternal conditions | Congenital Cytomegalovirus infection |
| 22 | 41.1 | Infectious | Chorioamnionitis (No agent) | Congenital pneumonia (S. Aureus) |
| 23 | 42.1 | Infectious | Chorioamnionitis (S. vaginalis) | Congenital pneumonia (No agent) |
| 24 | 32.2 | Infectious | Chorioamnionitis (No agent) | Congenital pneumonia (No agent) |
| 25 | 33.2 | Infectious | Chorioamnionitis (Prevotella spp) | Congenital pneumonia (No agent) |
| 26 | 34 | Infectious | Chorioamnionitis (S. agalactiae) | Intrauterine hypoxia |
| 27 | 38.6 | Pregnancy and fetal development | No maternal conditions | Intrauterine hypoxia (No agent) |
| 28 | 35.6 | Pregnancy and fetal development | Preeclampsia - Eclampsia | Intrauterine hypoxia |
| 29 | 33 | Pregnancy and fetal development | Preeclampsia - Eclampsia | Intrauterine hypoxia |
| 30 | 32 | Pregnancy and fetal development | Preeclampsia - Eclampsia | Intrauterine hypoxia |
| 31 | 37.1 | Pregnancy and fetal development | Preeclampsia - Eclampsia | Intrauterine hypoxia |
| 32 | 30.4 | Pregnancy and fetal development | Placental and umbilical cord alterations | Intrauterine hypoxia |
| 33 | 29.3 | Pregnancy and fetal development | Preeclampsia - Eclampsia | Intrauterine hypoxia |
| 34 | 33 | Pregnancy and fetal development | Preeclampsia - Eclampsia | Intrauterine hypoxia |
| 35 | 30.3 | Pregnancy and fetal development | Preeclampsia - Eclampsia | Intrauterine hypoxia |
| 36 | 28.6 | Pregnancy and fetal development | Preeclampsia - Eclampsia | Intrauterine hypoxia |
| 37 | 33.2 | Pregnancy and fetal development | No maternal conditions | Intrauterine hypoxia |
| 38 | 37.2 | Pregnancy and fetal development | Placental and umbilical cord alterations | Intrauterine hypoxia |
| 39 | 29 | Pregnancy and fetal development | Preeclampsia - Eclampsia | Intrauterine hypoxia |
| 40 | 24 | Pregnancy and fetal development | No maternal conditions | Congenital malformation |
| 41 | 41.3 | Pregnancy and fetal development | Preeclampsia - Eclampsia | Intrauterine hypoxia |
| 42 | 30.1 | Pregnancy and fetal development | Preeclampsia - Eclampsia | Intrauterine hypoxia |
| 43 | 35.6 | Pregnancy and fetal development | No maternal conditions | Intrauterine hypoxia |
| 44 | 34.5 | Pregnancy and fetal development | Preeclampsia - Eclampsia | Intrauterine hypoxia |
| 45 | 38.1 | Pregnancy and fetal development | No maternal conditions | Intrauterine hypoxia |
| 46 | 31.1 | Pregnancy and fetal development | No maternal conditions | Gastroschisis |
| 47 | 32 | Pregnancy and fetal development | Preeclampsia - Eclampsia | Intrauterine hypoxia |
| 48 | 40.1 | Pregnancy and fetal development | Placental and umbilical cord alterations | Intrauterine hypoxia |
| 49 | 36.5 | Pregnancy and fetal development | Preeclampsia - Eclampsia | Intrauterine hypoxia |
| 50 | 38.4 | Pregnancy and fetal development | Preeclampsia - Eclampsia | Intrauterine hypoxia |
| 51 | 36.2 | Pregnancy and fetal development | Preeclampsia - Eclampsia | Intrauterine hypoxia |
| 52 | 37.4 | Pregnancy and fetal development | Preeclampsia - Eclampsia | Intrauterine hypoxia |
| 53 | 32.2 | Pregnancy and fetal development | No maternal conditions | Intrauterine hypoxia |
| 54 | 32.2 | Pregnancy and fetal development | Preeclampsia - Eclampsia | Intrauterine hypoxia |
| 55 | 35.5 | Pregnancy and fetal development | Preeclampsia - Eclampsia | Intrauterine hypoxia |
| 56 | 39 | Pregnancy and fetal development | Placental and umbilical cord alterations | Intrauterine hypoxia |
| 57 | 31 | Pregnancy and fetal development | Placental and umbilical cord alterations | Intrauterine hypoxia |
| 58 | 28.4 | Pregnancy and fetal development | Preeclampsia - Eclampsia | Intrauterine hypoxia |
| 59 | 30.2 | Pregnancy and fetal development | Placental and umbilical cord alterations | Intrauterine hypoxia |
| 60 | 37.5 | Pregnancy and fetal development | Placental and umbilical cord alterations | Intrauterine hypoxia |
| 61 | 37.2 | Pregnancy and fetal development | Preeclampsia - Eclampsia | Intrauterine hypoxia |
| 62 | 36.4 | Pregnancy and fetal development | Preeclampsia - Eclampsia | Intrauterine hypoxia |
| 63 | 36.3 | Pregnancy and fetal development | Preeclampsia - Eclampsia | Intrauterine hypoxia |
| 64 | 36.2 | Pregnancy and fetal development | Preeclampsia - Eclampsia | Intrauterine hypoxia |
| 65 | 30.6 | Pregnancy and fetal development | Preeclampsia - Eclampsia | Intrauterine hypoxia |
| 66 | 33.2 | Pregnancy and fetal development | Preeclampsia - Eclampsia | Intrauterine hypoxia |
| 67 | 34.2 | Pregnancy and fetal development | Preeclampsia - Eclampsia | Intrauterine hypoxia |
| 68 | 27.5 | Pregnancy and fetal development | Preeclampsia - Eclampsia | Intrauterine hypoxia |
| 69 | 33 | Pregnancy and fetal development | Placental and umbilical cord alterations | Intrauterine hypoxia |
| 70 | 33.3 | Pregnancy and fetal development | Preeclampsia - Eclampsia | Intrauterine hypoxia |
| 71 | 34.5 | Pregnancy and fetal development | Preeclampsia - Eclampsia | Intrauterine hypoxia |
| 72 | 32.5 | Pregnancy and fetal development | Preeclampsia - Eclampsia | Intrauterine hypoxia |
| 73 | 29.2 | Pregnancy and fetal development | Preeclampsia - Eclampsia | Intrauterine hypoxia |
| 74 | 29 | Pregnancy and fetal development | Preeclampsia - Eclampsia | Intrauterine hypoxia |
| 75 | 31.2 | Pregnancy and fetal development | Preeclampsia - Eclampsia | Intrauterine hypoxia |
| 76 | 36.5 | Pregnancy and fetal development | Placental and umbilical cord alterations | Intrauterine hypoxia |
| 77 | 29.5 | Pregnancy and fetal development | Preeclampsia - Eclampsia | Intrauterine hypoxia |
| 78 | 34.6 | Pregnancy and fetal development | Preeclampsia - Eclampsia | Intrauterine hypoxia |
| 79 | 34.6 | Pregnancy and fetal development | Preeclampsia - Eclampsia | Intrauterine hypoxia |
| 80 | 31 | Pregnancy and fetal development | Preeclampsia - Eclampsia | Intrauterine hypoxia |
| 81 | 34.2 | Pregnancy and fetal development | Preeclampsia - Eclampsia | Intrauterine hypoxia |
| 82 | 37.5 | Pregnancy and fetal development | No maternal conditions | Fetal macrosomy |
| 83 | 35 | Pregnancy and fetal development | Preeclampsia - Eclampsia | Intrauterine hypoxia |
| 84 | 33.5 | Pregnancy and fetal development | Preeclampsia - Eclampsia | Intrauterine hypoxia |
| 85 | 28 | Pregnancy and fetal development | Preeclampsia - Eclampsia | Intrauterine hypoxia |
| 86 | 30.2 | Pregnancy and fetal development | Preeclampsia - Eclampsia | Intrauterine hypoxia |
| 87 | 38 | Pregnancy and fetal development | Placental and umbilical cord alterations | Intrauterine hypoxia |
| 88 | 40.1 | Pregnancy and fetal development | Preeclampsia - Eclampsia | Intrauterine hypoxia |
| 89 | 40.2 | Pregnancy and fetal development | No maternal conditions | Intrauterine hypoxia |
| 90 | 37.4 | Pregnancy and fetal development | Preeclampsia - Eclampsia | Intrauterine hypoxia |
| 91 | 28.4 | Pregnancy and fetal development | No maternal conditions | Intrauterine hypoxia |
| 92 | 31.1 | Pregnancy and fetal development | Diabetes mellitus in pregnancy | Intrauterine hypoxia |
| 93 | 33.1 | Pregnancy and fetal development | Preeclampsia - Eclampsia | Intrauterine hypoxia |
| 94 | 31.1 | Pregnancy and fetal development | Placental and umbilical cord alterations | Intrauterine hypoxia |
| 95 | 29.3 | Pregnancy and fetal development | Preeclampsia - Eclampsia | Intrauterine hypoxia |
| 96 | 35.2 | Pregnancy and fetal development | Preeclampsia - Eclampsia | Intrauterine hypoxia |
| 97 | 32.3 | Pregnancy and fetal development | Preeclampsia - Eclampsia | Intrauterine hypoxia |
| 98 | 35.2 | Pregnancy and fetal development | Placental and umbilical cord alterations | Intrauterine hypoxia |
